# Activation of p73 and induction of Noxa by DNA damage requires NF-kappa B

**DOI:** 10.18632/aging.100026

**Published:** 2009-02-18

**Authors:** Angel G. Martin, Jason Trama, Diane Crighton, Kevin M. Ryan, Howard O. Fearnhead

**Affiliations:** ^1^Apoptosis Section, NCI-Frederick, Frederick, MD 21702, USA; ^2^Tumour Cell Death Laboratory, Cancer Research UK Beatson Laboratories, Glasgow, G61 1BD, UK; ^3^current address: Fundacion Inbiomed, Paseo Mekeletegi 61, San Sebastian 20009, Spain; ^4^current address: National Centre for Biomedical Engineering Science, National University of Ireland, Galway, Ireland

**Keywords:** apoptosis, p73, NF-κB B, Noxa

## Abstract

Although the transcription factor NF-κB is most clearly linked to the inhibition of extrinsic apoptotic signals such as TNFα by upregulating known anti-apoptotic genes, NF-κB has also been proposed to be required for p53-induced apoptosis in transformed cells. However, the involvement of NF-κB in this process is poorly understood. Here we investigate this mechanism and show that in transformed MEFs lacking NF-κB (*p65*-null cells) genotoxin-induced cytochrome *c* release is compromised. To further address how NF-κB contributes to apoptosis, gene profiling by microarray analysis of MEFs was performed, revealing that NF-κB is required for expression of Noxa, a pro-apoptotic BH3-only protein that is induced by genotoxins and that triggers cytochrome *c* release. Moreover, we find that in the absence of NF-κB, genotoxin treatment cannot induce Noxa mRNA expression. Noxa expression had been shown to be regulated directly by genes of the p53 family, like p73 and p63, following genotoxin treatment. Here we show that p73 is activated after genotoxin treatment only in the presence of NF-κB and that p73 induces Noxa gene expression through the p53 element in the promoter. Together our data provides an explanation for how loss of NF-κB abrogates genotoxin-induced apoptosis.

## Introduction

Programmed cell death or apoptosis is of fundamental importance to cancer as it both limits tumorigenesis and is also triggered by many cancer chemotherapeutics [1,2]. Importantly, cancer cells often acquire mutations that compromise the apoptotic process, allowing these cells to both escape normal growth constraints and to become resistant to many anti-cancer drugs, resulting in the emergence of drug-resistant malignancies [3]. Thus discovering how apoptosis is regulated and why it fails in cancer is central to both understanding cancer progression  and  developing new therapies  to  counter chemo-resistant cancers. Many proteins involved in the apoptotic process have been identified [4], including proteins of the p53 family, a tumour suppressor whose function is compromised in many cancer cells.  p53 is a well established tumour suppressor and key regulator of apoptosis that is induced by both oncogenes and chemotherapeutic drugs (genotoxins) that damage DNA [5]. p53 induces apoptosis predominantly by increasing expression of genes of the Bcl-2 family, such as Bax, PUMA and Noxa, that trigger cytochrome *c* release from mitochondria into the cytosol [6]. Cytosolic cytochrome *c* binds to Apaf-1, which complexes with, and activates the initiator caspase-9. This leads to the activation of the effector caspase-3, resulting in cell death [7,8].

p53 is the first member of a closely related family which includes the proteins p63 and p73.  However, p63 and p73 are present in multiple isoforms and their role in tumour formation and apoptosis control is not as well defined as it is for p53.  Mouse knock out (K.O.) studies revealed unexpected functional diversity among these proteins.  p63 and p73 K.O. mice exhibit severe developmental abnormalities and no increased tumour formation, whereas p53 K.O.mice show no developmental defect but early appearance of tumours (for review see [9]).  Recent long-term studies in mice, however, support a direct role of p63 and p73 on tumour suppression. A recent K.O. mouse specific for the TA isoform of p73, however, shows an intermediate phenotype between the full p73 ^-/-^ and p53 ^-/-^ regarding tumour formation, supporting a role of p73 in tumour suppression [10]. Additionally, p63 and p73 can cooperate with p53 in tumour suppression [11].

Functionally, p73α and p73β closely resemble the biological activity of p53, including the ability to induce apoptosis.  p73β, and less efficiently p73α, bind to canonical p53 elements in the DNA and transactivate many p53 dependent promoters [12].

Recently, the transcription factor NF-κB, which clearly inhibits apoptosis induced by some death signals [13,14], was proposed to play a role in driving p53-mediated apoptosis in transformed cells [15]. It has also been suggested a proapoptotic role in cerebral ischemia through p65 (RelA) containing complexes [16,17]. The canonical NF-κB complex is a heterodimer of p50 and p65, which accounts for the majority of the NF-κB complexes found in non-immune cells. NF-κB is activated by a variety of signals, including pro-inflammatory and stress factors, that cause phosphorylation of the IκB inhibitory proteins by the IκB-kinase complex. Phosphorylation marks IκB for ubiquitinylation and proteasomal degradation, allowing NF-κB complexes to localize to the nucleus where they affect transcription (for review see [18]).  The idea that NF-κB is pro-apoptotic is, however, controversial for several reasons. Elevated NF-κB activity is associated with increased tumorigenesis [19] and decreasing its activity can inhibit tumorigenesis [20]. Consistent with these observations is the fact that several anti-apoptotic genes are NF-κB targets and NF-κB activation can protect cells from apoptosis, therefore increasing oncogenic potential [21-23]. Indeed, NF-κB activation can reduce p53 stabilization triggered by chemo-therapeutics [24]. However, the effect of NF-κB on tumorigenesis and apoptosis appears to be context-dependent because inactivation of NF-κB can promote tumorigenesis [25] and prevent both p53- [15] and chemotherapy-induced apoptosis [26,27]. In addition, two genes deregulated in human tumours, β-catenin and HSCO, inhibit NF-κB activity by sequestering NF-κB in the cytosol, blocking Fas-induced [28] and p53-induced apoptosis [29]. NF-κB is also implicated in apoptosis induced by growth factor withdrawal, viruses and ischemia [30-34].  Why NF-κB might be required for p53-induced apoptosis is unknown. To further evaluate the idea that NF-κB is pro-apoptotic, we chose to investigate genotoxin-dependent apoptosis in cells lacking p65.  By using a combination of approaches, we show that in the absence of p65, DNA damage-induced expression of a pro-apoptotic BH3-only protein, Noxa, is compromised.  Although p53 present in the immor-talized p65 null cells used in our study is a non-functional mutant, our experiments show that DNA damage-induced activation of p73 depends on the presence of p65.  Furthermore, p73 regulates the expression of Noxa through the p53 element in its promoter.  Thus in the absence of p53, NF-κB controls the DNA damage-induced cell death through the activation of p73 induced Noxa expression.

## Results

**Figure 1.**
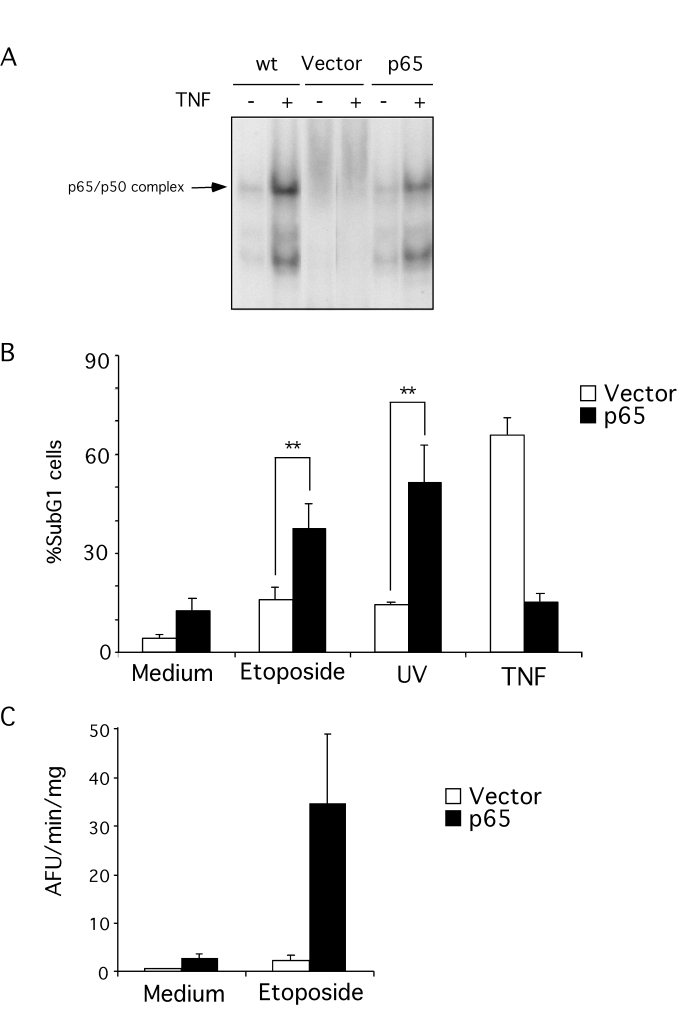
Apoptosis resistance in p65 null MEFs. (**A**) retrovirus-mediated reconstitution of p65 null MEFs with p65 restores NF-κB function as measured by EMSA. Wild type (wt), p65 null (vector) and p65 null reconstituted MEFs were stimulated with 10 ng/ml TNFα for 6 hr. Nuclear proteins were extracted and equal amounts of extract incubated with a radio-labeled NF-κB consensus probe. (**B**) p65 null cells are resistant to genotoxin-induced apoptosis. Cells were treated with 10 μM etoposide or 5 mJ UV-irradiation for 18 hr. Floating and attached cells were then collected and stained with propidium iodide (PI). DNA content was analyzed by flow cytometry. Results are presented as percentage of cells with sub-G_1_ DNA content. The data shown represent the mean and SEM of three independent experiments. **statistically significant by student t-test analysis (p<0.05). (**C**) S-100 extracts from p65 null (vector) and reconstituted cells (p65) treated with 10 μM etoposide were used to assess caspase activity by cleavage (arbitrary fluorescence units per minute [AFU/min]) of the fluorogenic substrate, Ac-DEVD-afc. The data shown represent the mean and SEM of three independent experiments.

p65 null MEFs are resistant to genotoxin-induced death To address how NF-κB deficiency alters p53-dependent apoptosis in transformed cells we examined the ability of p65 null MEFs transformed with the adenoviral oncogene E1A to undergo apoptosis induced by genotoxic agents. As a control for these studies NF-κB function was reconstituted by retroviral gene transfer of p65 (Figure 1A). To control for this manipulation p65 null MEFs were mock infected with an empty retroviral vector.  Thus these two cell types differ only in p65 expression and are a closer genetic match than available wild type MEFs. When the sensitivity to apoptosis induced by genotoxic agents of the p65 null and reconstituted MEFs was compared cells lacking p65 were resistant to both a topoisomerase inhibitor (etoposide) and UV-irradiation, two DNA-damaging agents that activate cell death. However, reconstitution of p65 greatly increased sensitivity to induction of apoptosis by these agents.  Apoptosis was assessed by the appearance of cells with hypodiploid DNA content (Figure 1B) and also by activation of caspases (Figure 1C), therefore two criteria confirmed that loss of p65 confers resistance to genotoxin-induced apoptosis. In contrast, p65 null cells are extremely sensitive to TNFα induced apoptosis, while p65 reconstitution confers resistance, consistent with the p65-dependent activation of anti-apoptotic genes by TNFa [13,41-47].

### p65 null MEFs retain functional apoptotic machinery

**Figure 2.**
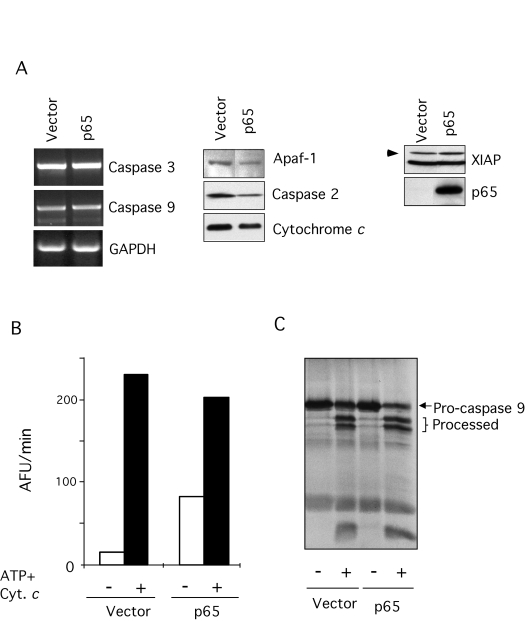
Characterization of the apoptotic machinery in p65 null MEFs. (**A**) Expression of several key components of the apoptotic machinery in p65 null and reconstituted cells was compared. Expression of Apaf-1, caspase-2, cytochrome *c*, and XIAP was detected by immuno-blot. Expression of caspase-3 and -9 was assessed by RT-PCR from total RNA extracted from the cells as indicated. (**B**) S-100 extracts from p65 null (vector) and reconstituted cells (p65) were incubated with 1 mM ATP and 1 μM equine cytochrome *c* at 37 °C for 1 hr. Caspase activity was then assessed by cleavage (arbitrary fluorescence units per minute [AFU/min]) of the fluorogenic substrate, Ac-DEVD-afc. (**C**) caspase-9 processing by autoradiography. S-100 extracts were incubated under the conditions described above with *in vitro* translated caspase 9 and subjected to SDS-PAGE.

To investigate what stage of the genotoxin-induced pathway was compromised by loss of p65, we first checked the expression of several key components of the apoptotic pathway (Figure 2A).  mRNA level for caspases-3 and -9 was assessed by RT-PCR due to the lack of suitable antibodies, while other proteins implicated in either effecting or inhibiting apoptosis were assessed by immunoblot. In addition, levels of caspase-2, which has recently been implicated as playing an essential initiating role in genotoxin-dependent apoptosis [48], was also assessed (Figure 2A). In no instance was there a detectable difference between the null and reconstituted cells indicating that the loss of p65 did not alter the expression of these death machinery components. To confirm that the cell-death machinery was competent we utilized a cell-free system for quantitation of caspase activation. In this system caspases are activated by the addition of cytochrome *c*, triggering Apaf-1-dependent activation of caspase-9 and -3 [49]. Therefore, to test the functionality of the death machinery, cytochrome *c* was added to S-100 extracts from p65 null or reconstituted cells and caspase activation was assessed. As shown in Figure 2B and C, extracts derived from both cell types were equally capable of caspase activation, demonstrating that p65 null cells retained functional death machinery downstream of cytochrome *c* (Figure 2B and C).

### Cytochrome *c* release is impaired in p65 null cells

**Figure 3.**
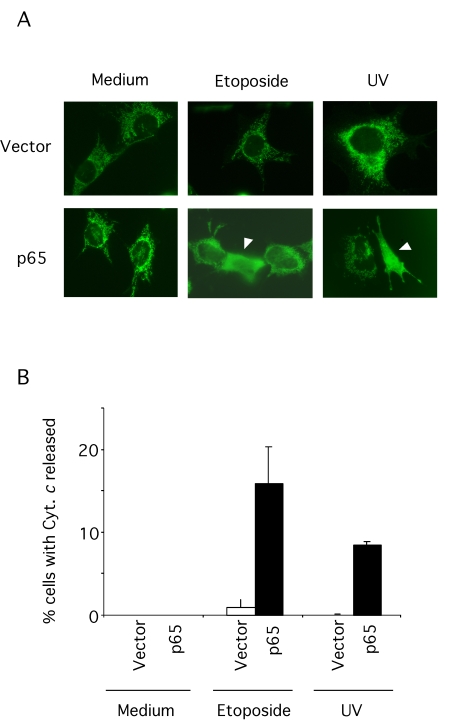
Cytochrome *c* release in p65 null cells. (**A**) p65 null (vector) and reconstituted cells were treated with 10 μM etoposide or 5 mJ UV-irradiation in the presence of the caspase inhibitor, zVAD-fmk (50 μM) for 18 hr and then fixed and stained with a specific antibody for native cytochrome *c***. **An Alexa Green coupled secondary antibody was used to reveal the localization of cytochrome *c. (***B**) Results are expressed as percentage of cells showing cytosolic cytochrome *c*.

The next step upstream in the p53-induced apoptotic pathway is mitochondrial release of cytochrome *c*, controlled by the interaction between Bcl-2 family members. Therefore, we compared the sub-cellular localization of cytochrome *c *in p65 null and reconstituted cells after etoposide and UV-irradiation.  Morphological changes triggered by caspases during apoptosis make it very difficult to assess the sub-cellular localization of proteins. To allow accurate quantitation of cells with cytoplasmic cytochrome *c*, the apoptotic consequences of cytochrome *c* release were prevented with the caspase inhibitor z-VAD-fmk. In reconstituted cells, both stimuli induced cytoplasmic localization of cytochrome *c* after 24 hr treatment (Figure 3). The percentage of cells showing released cytochrome *c* was lower than the incidence of apoptosis shown in Figure 1B. However, in Figure 1B the percentage of apoptotic cells in the total population, both detached and attached, was assessed. While immuno-localization of cytochrome *c* was assessed only in those cells that remained attached to the culture plates, explaining why fewer apoptotic cells were detected in this assay. In contrast, very few p65 null MEFs showed cytoplasmic cytochrome *c* localization in response to genotoxins. These results suggest that the absence of p65 impaired p53-dependent death at or before cytochrome *c* release from the mitochondria.

## Gene profiling of p65 null mefs reveals lack of noxa expression

Since p65 is a transcription factor, we reasoned that there may be one or more genes whose expression is compromised in p65 null MEFs and whose function is necessary for p53-dependent death, most likely controlling cytochrome *c* release from mitochondria.

**Figure 4.**
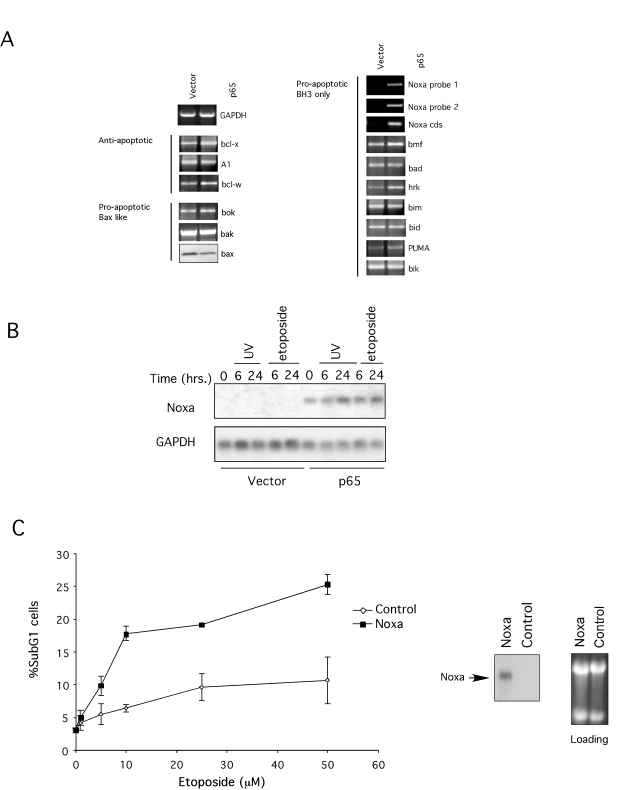
Expression of Bcl2 family members. (**A**) RT-PCR of bcl2 family members. cDNA was prepared from total RNA from p65 null (vector) and reconstituted cells (p65). Specific oligonucleotides for each gene (and three pairs for Noxa) were used to determine expression. GAPDH expression was used as a control. Bax expression was detected by immunoblot. (**B**) Northern Blot for Noxa after genotoxic treatments. Total RNA was extracted from p65 null and reconstituted cells after treatment with 10 μM etoposide or 5 mJ UV-irradiation for the times indicated. Expression of Noxa, and GAPDH as control, was revealed by blotting with specific radio-labeled probes. (**C**) Expression of Noxa sensitizes p65 null MEFs to genotoxic agents. Cloned murine Noxa was introduced into p65 null cells by retroviral transfer and sensitivity to etoposide and UV-irradiation compared. Noxa cloned in the anti-sense orientation was used as a control. After selection cells were treated with 10 μM etoposide or 5 mJ UV-irradiation for 24 hr and apoptosis assessed by flow cytometry as described in Figure 1. Results are representative of three different viral clones for both control and Noxa. Northern blotting confirmed Noxa expression.

Therefore, we compared the expression profile of p65 null versus p65 reconstituted cells using gene microarray analyses. Strikingly, expression profiling revealed that one of the genes upregulated by p65 expression was Noxa, a pro-apoptotic BH3-only protein of the Bcl-2 family [50] (Table 1). Previously, this protein was reported to be induced by p53 and to be required for p53-induced death by controlling cytochrome *c* release [38,51], making it a candidate for immediate study. Of the other Bcl-2 family members screened none belonged to the top most differentially regulated genes (ratio p65 reconstituted/null >1.7; Table 1). To validate the microarray data RT-PCR assays were performed to compare the expression level of several other Bcl-2 family members and the ones not represented in the array. As shown in Figure 4A, Noxa expression was absent in p65 null cells but expression was restored by re-introducing p65 (Figure 4A). In contrast, expression of other Bcl-2 family member was not impaired in p65 null cells. Likewise, immunoblot analysis showed Bax expression was not altered by the p65 status of the cells (Figure 4A). To assess whether Noxa expression could be induced by genotoxins, levels of Noxa mRNA from etoposide or UV-irradiation treated p65 null and reconstituted cells were evaluated by Northern blot. Noxa mRNA could not be detected in untreated p65 null cells but was present in the p65 reconstituted cells, consistent with the microarray analysis (Figure 4B). Moreover, Noxa mRNA expression could be induced by etoposide or UV-treatment in the reconstituted cells but not in the p65 null cells (Figure 4B). Thus, p65 is necessary for Noxa expression and for genotoxin-dependent induction of Noxa.

**Table 1.** Comparison of expression of several genes present in the array. Expression of caspases is shown as constitutive genes. Average Ratio (n=5)

P65/vector	stdev	Gene	
0.7	0.2	Bad	Bcl-2 family
0.6	0.1	Bag1	
1.2	0.3	Bag3	
0.7	0.1	Bak1	
0.9	0.2	Bax	
0.6	0.1	Bcl2l	
1.4	0.5	Bcl2l10	
1.4	0.5	Bcl2l2	
1.0	0.1	Biklk	
1.0	0.1	Bnip2	
0.6	0.2	Bnip3l	
0.8	0.3	Bok	
1.9	0.1	Noxa	
0.8	0.3	Casp1	Caspases
1.3	0.2	Casp2	
0.8	0.3	Casp3	
1.2	0.2	Casp6	
1.1	0.2	Casp7	
1.4	0.4	Casp8	
0.7	0.1	Casp9	
1.2	0.5	Casp11	
0.9	0.4	Casp12	

Noxa has been shown to be necessary for p53-dependent apoptosis [38,51], however, it is not clear whether expression of Noxa is sufficient to explain sensitivity to genotoxic agents. To address this issue Noxa was re-introduced in p65 null cells by retroviral transfer. Expression of the exogenous Noxa was confirmed by Northern blot analysis (Figure 4C). As depicted in Figure 4C, reconstitution of Noxa expression was not sufficient to promote death; however, Noxa expressing cells were more sensitive to genotoxic treatment than control cells. Thus, expression of Noxa in p65 null cells restored sensitivity to genotoxic agents.

### p53 is mutant in p65 null and reconstituted cells

Noxa has been shown to be necessary for p53-dependent death and its expression is indeed induced by p53 [38,51].  MEFs and, particularly, transformed cell lines derived from MEFs, very frequently acquire mutations in the p53 tumour suppressor.  A trivial explanation of our data is that the p65 null cells but not the p65 reconstituted cells had acquired a p53 mutation during serial passage and immortalization, thus explaining the lower apoptotic sensitivity of p65 null cells to DNA damaging agents.  To test this possibility, the DNA sequence of the p53 gene from both p65 null and reconstituted cells was compared.  Both cell lines showed identical sequence for the p53 gene, excluding the possibility that a p53 mutation would explain the observed difference in sensitivity to genotoxins. However, these data revealed that p53 was mutant: there was a silent mutation at codon 82 (c to t at base 246) and a missense mutation at codon 275 (c to g at base 824) that results in a Proline to Arginine substitution.  This position corresponds to codon 278 in human p53 within the DNA binding domain, an extremely well conserved region.  This particular Pro278Arg mutation has been found in human tumours although the functionality of this mutant had not been previously tested.  Moreover, no wild type allele was detected in our sequencing and Southern Blotting revealed that both p65 null and reconstituted cells had only one copy of p53 (data not shown).

**Figure 5.**
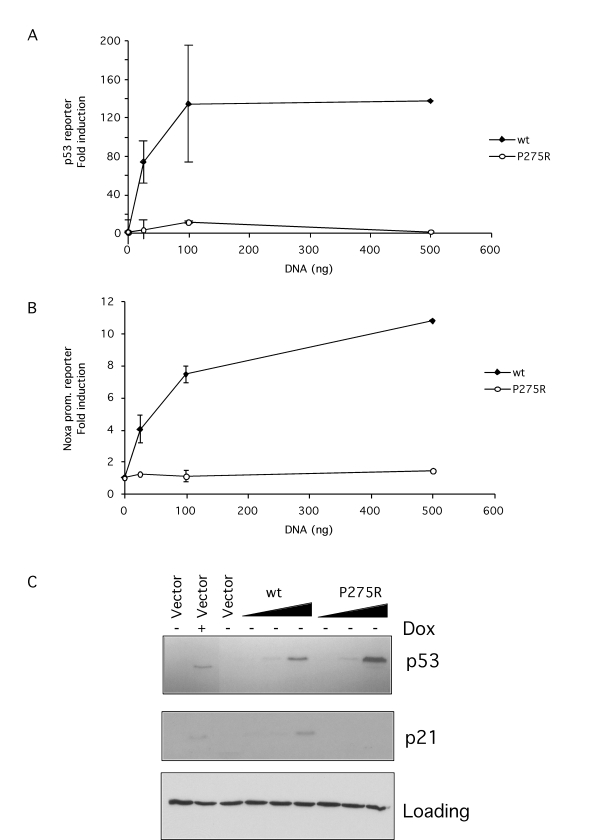
p53 in p65 null and reconstituted cells is a non-functional mutant. (**A-B**) 0.5 μg of PG13-Luc p53 luciferase reporter (**A**) or Noxa promoter luciferase reporter (**B**) were co-transfected into SaOS-2 cells along with increasing amounts of the wild type or P275R mutant p53 vectors. 48 hr after transfection luciferase activity was compared.  Results are expressed as fold induction above mock (empty pcDNA3 vector) control.  (**C**) p53 P275R or wild type expression was demonstrated by immunoblotting in extracts derived from SaOS-2 p53 tet-on cells transfected as described for A-B. As a control, p53 was induced by doxocycline treatment. Endogenous p21 induction was assessed by immunoblotting from the same extracts.  A non-specific band detected with the p21 antibody was used as loading control.

To test the function of this mutant p53 its ability to activate a reporter gene was tested.  Mutant p53 was first cloned from p65 null cells by RT-PCR.  To control for the activity of this mutant codon 275 was reverted to wild type by site directed mutagenesis. Mutant or wild type p53 was then expressed in the p53 null cell line SaOS-2 along with a PG13 p53-responsive reporter construct (Figure 5A) or the Noxa promoter -183 to +146 in front of the luciferase reporter gene construct (Figure 5B).  The data clearly showed that wild type p53 activated both the p53 reporter and the Noxa promoter reporter constructs while P275R mutant failed to do so.  Immunoblotting showed that this lack of activity could not be explained by differences in p53 expression (Figure 5C). Expression of a well known p53 target, p21, was also assessed by immunoblotting and again, while wild type p53 induced expression of p21, mutant p275R failed to do so (Figure 5C).  In further experiments the ability of the P275R mutation to interfere with wild type p53 was tested. However, no interference was observed (data not shown) indicating that the P275R mutation, unlike some other p53 mutations, did not generate a dominant negative p53.

Thus p53 status cannot explain the difference in sensitivity of the p65 null cells and reconstituted.  Moreover, genotoxin induced death and induction of Noxa expression in these cells is p53 independent.

### Control of Noxa expression and apoptosis induction by p73

p73 is a member of the p53 family that has been shown to promote apoptosis and to activate p53 target genes through the p53 elements in their promoters [52-56]. Recently, E1A activation of p73 and induction of Noxa expression in the absence of p53 in an osteosarcome cell line has been shown [57].  To assess whether Noxa can be induced by p73, a reporter approach was used.

**Figure 6.**
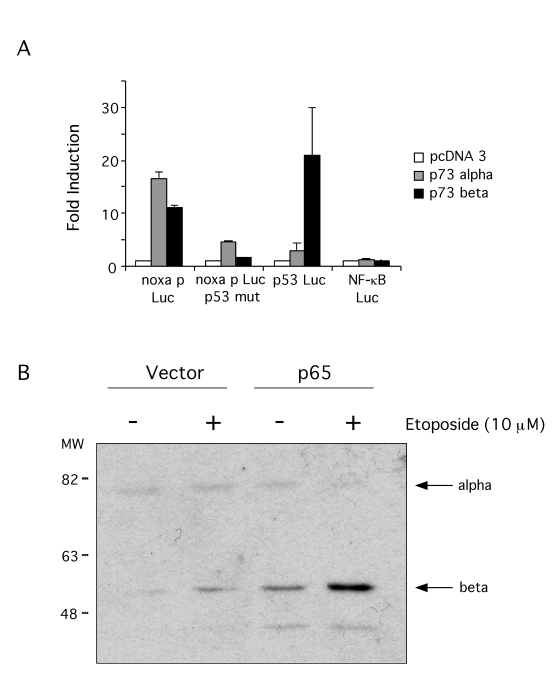
p73 induces Noxa promoter. (**A**) SaOS-2 cells were transfected with 1 μg of pcDNA3 control vector, p73α or p73β expression vectors along with the following luciferase reporter plasmids: Noxa promoter reporter, Noxa promoter p53 mutant reporter, PG13-Luc p53 reporter or NF3TK-Luc NF-κB reporter (as control).  48 hr after transfection luciferase activity was compared.  Results are expressed as fold induction above mock (empty pcDNA3 vector) control.  (**B**) p73 activation in p65 null and reconstituted cells. Cells were treated with 10μM etoposide for 24 hr and p73 levels determined by immunoblotting with a pan-p73 antibody.

p73 expression vectors were transiently transfected into SaOS-2 cell line along with the Noxa promoter reporter.  Both p73α and p73β activated the expression of the reporter and they also activated expression of the PG13 p53 reporter (Figure 6A).  To test if p73 was activating the Noxa promoter through the p53 element, a mutant promoter was used were the p53 element had been eliminated.  Neither p73α nor p73β activated the expression of this reporter indicating that Noxa expression by p73 uses the p53 element in its promoter, consistent with previous reports.  As a control, p73 did not activate transcription of a NF-κB dependent reporter (Figure 6A).  A p73 inducible SaOS-2 cell line was also used to demonstrate Noxa reporter induction by p73. Induction of p73 expression by Doxicycline indeed activated Noxa reporter but failed to activate the mutant promoter for the p53 element (data not shown), thus corroborating that p73 controls Noxa promoter.

Having confirmed that p73 can control Noxa promoter, we tested next if DNA damage can induce p73 in p65 null and reconstituted cells.  Cells were treated with etoposide for 24 hr and p73 levels assessed by immuno-blotting. This revealed the induction of a 52 kDa protein, consistent with the p73β isoform (Figure 6B).  While induction was seen in both cell types, the levels of p73 in the p65 reconstituted cells were markedly higher than in the p65 null cells.  p73 levels in etoposide treated p65 null cells were comparable to basal levels in the reconstituted cells, which were significantly induced by etoposide treatment.

### Dominant negative p73β blocks genotoxin-induced apoptosis and Noxa expression

**Figure 7.**
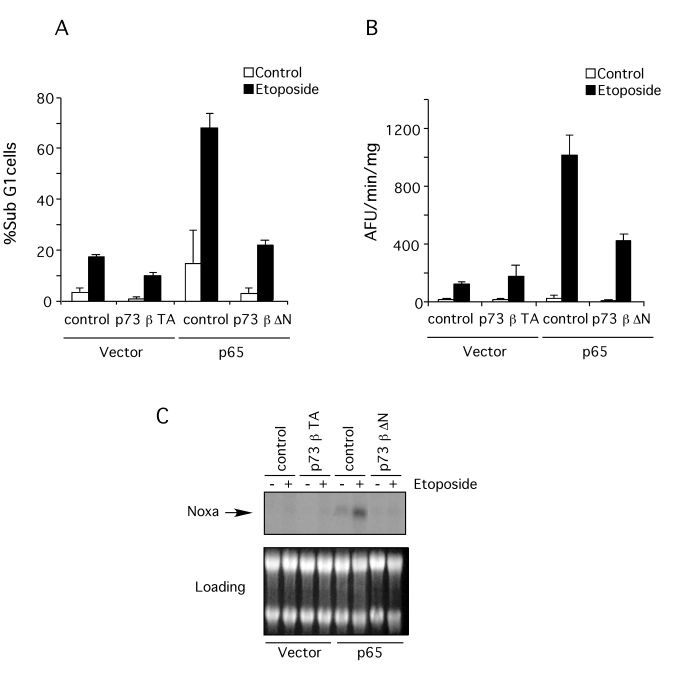
Dominant negative p73β blocks apoptosis induction and Noxa expression. p73β wild type was introduced into p65 null cells and p73β dominant negative was introduced into p65 reconstituted cells by retroviral transfer.  Then apoptosis induction and Noxa expression was assessed after treatment with 10 μM etoposide for 18 hr. (**A**) Floating and attached cells were then collected and stained with propidium iodide (PI). DNA content was analyzed by flow cytometry. Results are presented as percentage of cells with sub-G_1_ DNA content. The data shown represent the mean and SEM of three independent experiments. (**B**) S-100 extracts from p65 null (vector) and reconstituted cells (p65) were used to assess caspase activity by cleavage (arbitrary fluorescence units per minute [AFU/min]) of the fluorogenic substrate, Ac-DEVD-afc. The data shown represent the mean and SEM of three independent experiments. (**C**) Northern Blot for Noxa expression. Total RNA was extracted from p65 null (vector) and reconstituted (p65) cells after treatment with etoposide. Expression of Noxa was revealed by blotting with a specific radio-labeled probe. As loading control the ethidium bromide stained gel previous to transfer onto membrane is shown.  This blot is representative of three independent experiments.

The previous data suggest that the reason p65 null cells are less sensitive to DNA damage is the failure to induce sufficiently high levels of p73.  To test this possibility, a dominant negative form of p73β (ΔN-p73β) was expressed in the p65 reconstituted cells by retroviral transfer and the level of apoptosis following etoposide treatment determined.  Apoptosis induction, assessed both by hypodiploid DNA content (Figure 7A) and caspase activation (Figure 7B), showed that dominant negative p73β effectively blocked apoptosis.  This result indicates that p73 activation is necessary for DNA damage-induced apoptosis in this context.  Over-expression of wild type p73β in the p65 null cells, however, did not induce apoptosis.  Moreover, etoposide treatment of p65 null cells expressing wild type p73β did not cause apoptosis (Figure 7A and B), suggesting that although necessary, p73 expression was not sufficient for etoposide-induced apoptosis in the absence of p65.

We used Northern blotting in order to test whether p73β controls Noxa expression in p65 null cells.  As shown in Figure 7C, over-expression of wild type p73β did not restore Noxa expression in p65 null cells, even after etoposide treatment.  However, expression of a dominant negative  form of p73β in p65 reconstituted  cells successfully blocked expression of Noxa in these cells and prevented Noxa induction by etoposide treatment.  These results are in accordance with the effect of dominant negative p73β expression in apoptosis induction indicating that Noxa expression is the key regulator of apoptosis induced by genotoxins in the absence of p65.

## Discussion

DNA damage-induced apoptosis has been reported to require NF-κB [15], although which step of the apoptotic process is NF-κB dependent and which gene(s) are involved was not known. To investigate this issue, we characterized p65-dependent apoptosis using p65 null MEFs, which are resistant to genotoxin-induced apoptosis. These experiments uncovered a defect in the release of cytochrome *c* from mitochondria. We then performed expression profiling to identify candidate mediators of this effect.  Our microarray analysis showed that Noxa -which acts to trigger cytochrome *c* release and is a known DNA damage-induced gene- was missing in the absence of p65.  Noxa is a pro-apoptotic BH3-only member of the Bcl2 family that binds and blocks the anti-apoptotic function of Bcl-2 protein, thus promoting cytochrome *c* release from the mitochondria and death [38]. Therefore, lack of Noxa expression was likely to explain the apoptotic defect in p65 null cells. Subsequent experiments confirmed that p65 was indeed required for genotoxin-dependent induction of Noxa mRNA. Importantly, re-introducing Noxa into p65 null cells sensitized them to apoptosis induced by genotoxic agents. Interestingly, despite Noxa being necessary for genotoxin-induced apoptosis, ectopic expression of Noxa alone did not induce apoptosis. These findings suggest that Noxa expression alone is not sufficient for cell death and other factors may be required.

Noxa is known to be a transcriptional target of p53, a tumour suppressor that is activated by both oncogenes and genotoxic chemotherapeutic drugs.  The experi-ments described in this work were performed using transformed and immortalized MEFs, which frequently acquire p53 mutations during cell culture.  Clearly, our interpretation would be invalidated if the p65 reconstituted cells acquired a mutation that com-promised p53 but the p65 null cells did not.  When we sequenced p53 we found that both cell types had identical p53 sequence, excluding the possibility that a differential p53 function explained the p65 resistant phenotype.  However, p53 had acquired two mutations, a silent mutation in codon 82 and a substitution in codon 275, from Proline to Arginine.  This position is within the DNA binding domain of p53 and corresponds to P278R in human p53, a mutation that is found in a subset of human tumours.  The effect on p53 function of this mutation was unknown. Our analysis showed that this mutation compromised p53 ability to transactivate target genes, including Noxa.  Therefore p53 is not responsible for Noxa induction in our cells.

p63 and p73 are proteins functionally and structurally related to p53, constituting a family of related transcription factors.  The overall structure of the three proteins is quite similar, producing remarkably similar effects when over-expressed in cells [58,59].  E1A transformed MEFs deficient in both p63 and p73 are resistant to genotoxin-induced apoptosis, even in the presence of p53, therefore suggesting that these three genes might act together or p63/p73 act in an independent pathway to activate DNA damage-induced apoptosis [60].  Moreover, p73 activation is induced by a subset of DNA damaging drugs and blocking its function with a dominant negative mutant or siRNA led to apoptosis resistance of transformed human cell lines, irrespective of p53 status [61]. Activation of p73 alone can induce apoptosis suggesting a pro-apoptotic role on its own.  Several of the p53 dependent genes involved in apoptosis have been demonstrated to be significantly regulated by p73, such as Bax, DR5 and PUMA [52], and p53 binding to the promoters of its pro-apoptotic targets PERP, Noxa and Bax required the presence of p73 and/or p63 [60].  Moreover, Noxa was recently shown to be a p73 target to trigger E1A-induced apoptosis in p53 deficient cells [57]. Consequently, we  investigated the role of p73 in DNA damage-induced apoptosis in the p65 null cells.  Our data showed that activation of p73 by genotoxins was compromised in the absence of p65.  By using a dominant negative p73 mutant we demonstrated that genotoxin-induced apoptosis relies on p73 activation and importantly, that p73 activation is required for Noxa expression in our cells.  We also provide, for the first time, formal proof that Noxa is regulated by p73 at the promoter level through the p53 element.

How NF-κB participates in this process is unclear.  The NF-κB transcription factor is widely accepted as an anti-apoptotic factor [62] and several anti-apoptotic genes (for review see [22]) are known to be activated by NF-κB following treatment with TNFα. Moreover, the embryonic lethality in p65 knock-out mice is caused by extensive TNFα induced apoptosis in the liver [63]. NF-κB activation in tumor cell lines by chemotherapy has been reported and inhibition of NF-κB activation can enhance apoptosis induced by chemotherapy in a xenograft model of tumorigenesis [64,65]. These are strong data consistent with an anti-apoptotic role for NF-κB.  However, in other situations NF-κB appears to be pro-apoptotic [15-17,31-34,66]. As suggested by Blagosklonny [67], cellular responses should be defined in molecular terms where the same signalling pathways may participate in different, and often contradictory, end-points (in our case, induction of apoptosis vs. survival). Upstream signaling is initiated simultaneously and the cell translates it according to cellular context. Therefore NF-κB may act as a stress response transcription factor whose effect on a cell is context-dependent.  There may also be mechanistic differences between the pro-apoptotic activity and anti-apoptotic activity of NF-κB; suppression of steady state but not stimulus-induced NF-κB activity inhibits Alphavirus-induced apoptosis [68].  This is consistent with our observations that TNFα, a well-known activator of NF-κB through the canonical pathway, had no effect on Noxa expression, even though a NF-κB control reporter was activated (data not shown).

In our system, the pro-apoptotic effect of NF-κB depends on the activation of p73.  How this is accomplished is not clear.  p73 activation is mediated in part by protein stabilization, as it is for p53, since proteasome inhibitors stabilize the protein [69].  In contrast to p53, however, p73 degradation is not mediated by MDM2, although p73 binds to MDM2 and blocks its transcription promoting activity.  p73 stabilization and activation by genotoxic stress is also associated with p73 phosphorylation.  Several kinases have been implicated in this step. Thus following γ-irradiation c-Abl phosphorylates p73 at Tyr99 activating p73 and inducing apoptosis [70,71]. Phosphorylation at Tyr120 and Tyr240 were also shown [72].  The checkpoint kinases, CHK1 and CHK2, which are activated following DNA damage may also play a role, controlling p73 mRNA induction [73]. Aurora Kinase A regulates p73 dependent apoptosis in p53 deficient cell lines [74].  It is possible that absence of p65 DNA damage fails to activate p73 because the activity of one or all of these kinases is compromised in the absence of p65. We showed that ectopic expression of p73β alone in the absence of p65 was not sufficient to reinstate expression of Noxa or restore apoptosis sensitivity.  These data indicate that the absence of p65 compromises other steps in genotoxin-induced apoptosis in addition to p73. The simplest model is that p65 is required for the DNA-damage induced signalling pathways upstream of p73.

Since inhibition of NF-κB is currently being explored as a way of potentiating anti-cancer therapy [21,75] it is essential to define specifically where and when NF-κB shows a preferentially pro- or anti-apoptotic face. The observation that NF-κB controls expression of Noxa in the absence of functional p53, and loss of p53 function occurs in >50% of human tumours, suggests that in some contexts inhibition of NF-κB may compromise, rather than enhance, the efficacy of conventional anti-cancer therapy.

## Materials and methods

Plasmids, reagents and antibodies. Murine p65 was cloned into the EcoRI site in the retroviral vector pWZL-Hygro.  Murine Noxa was cloned from cDNA made from p65 reconstituted MEFs as a HA tagged fusion gene into the BamHI/EcoRI site of pcDNA3.1+ and the retroviral vectors pWZL-Blast and pBabe-Puro. Mutant P275R p53 was obtained by RT-PCR from immortalized p65 null cells and cloned directly into pcDNA3-TOPO (Invitrogen). Wild type p53 was generated by reverting the P275R mutation using the Quick-change site directed mutagenesis kit (Stratagene) following manufacturer's instructions. Expression vectors for p73α, DN-p73α, p73β and DN-p73β were kindly provided by Prof. G. Melino (University of Leicester, UK) and were subcloned into pWZLBlast retroviral vector.  Recombinant mouse tumor necrosis factor α (TNFα) and etoposide were purchased from Sigma. For immunoblotting we used antibodies against human Noxa (Imgenex), Bax, caspase 2, p21 (Santa Cruz Biotechnology), anti-HA (kindly provided by Dr P. Kaldis, NCI-Frederick, Frederick, MD), Apaf-1 (Alexis), cytochrome *c* (BD-Pharmingen), β-actin (Abcam), XIAP (BD-Transduction Laboratories), p53 (supernatant from culture of the DO-1 hybridoma), p73 (provided by Prof. G. Melino, University of Leicester, UK) and p65 (kindly provided by Dr N. Rice, NCI-Frederick, Frederick, MD).

Cells, transfection and retroviral gene transfer. Mouse fibroblasts and human cancer cells were grown in Dulbecco's modified minimal essential medium (DMEM, Gibco) supplemented with 10% fetal bovine serum (FBS, Gibco) at 37 °C in 10% CO_2_. Cells were transfected with the Lipofectamine Plus reagent (Gibco) in accordance with the manufacturer's instructions and washed after 3 hr incubation before adding fresh DMEM+10% FBS and incubating for a further 18 hr. For retoviral transfer, viral vectors were transfected into the Phi-NX ecotropic packaging cell line and after 24 hr the transfection culture medium was filtered and added to p65 null MEFs.  Infected cells were selected using the appropriate antibiotic.

Electro-Mobility Shift Assay (EMSA). Preparation of nuclear extracts was previously described [35]. The binding reaction consisted of 10 μg of extracted nuclear protein and 5 μg of poly dI-dC (Roche) in a total reaction volume of 10 μl containing 6 mM MgCl_2_. This mixture was then incubated at room temperature for 10 minutes, after which 2 μl (50,000 cpm) of the NF-κB consensus oligonucleotide (Promega), end-labeled with [^32^P]-γ-ATP (specific activity = 3,000 Ci/mmol; Amersham), was added. A control reaction mixture containing a 100-fold molar excess of non-radioactive NF-κB oligonucleotide was used to verify the specificity of the binding reaction. After incubation at 4˚C for 15 minutes the reaction mixtures were run on a 5% PAGE. After drying, the gels were subjected to autoradiography.

Extract preparation and caspase activity assay. 2 x 10^8^ p65 and reconstituted MEFs were used to prepare S-100 extracts as described [36].  Briefly, cells were harvested by trypsinization and washed in PBS. Cells were resuspended in 10 ml of extract buffer (50 mM PIPES, pH 7.0, 50 mM KCl, 5 mM EGTA, 2 mM MgCl_2_, 1 mM DTT, 2 μg/ml each of Leupeptin, Chymostatin, Antipain and Pepstatin A, 10 μg/ml Cytochalasin B and 100 μM PMSF), centrifuged and excess buffer immediately removed. Cells were then lysed by three freeze-thaw cycles in liquid nitrogen and centrifuged at 100,000 x *g* for 60 minutes to obtain an S-100 extract (≈30 mg/ml protein by Bradford assay). For caspase activity assessment, 30 μg of cell extract were used to determine conversion of the fluorogenic caspase substrate Ac-DEVD-afc (Biomol).  For caspase activation, equine cytochrome *c* (1 μM; Sigma) was added and extracts incubated at 37 ˚C for 60 minutes with 1 mM ATP as indicated. After this time caspase activity was determined using a Cytoflour 2000 flourimeter.

Cytochrome *c* immuno-localization. p65 null and reconstituted MEFs were grown on glass coverslips prior to treatment with etoposide (10 μM) or UV-irradiation (5 mJ). 18 hr later cells were fixed in 2% formaldehyde and permeabilized with 0.2% Triton. Fixed cells were incubated with an anti-native cytochrome *c *antibody. A secondary antibody coupled to Alexa Green (Molecular Probes) was used to detect cytochrome *c*.

Flow cytometry. Floating cells were recovered and pooled with adherent cells harvested by trypsinization. Cells were resuspended in PBS containing 1% Triton, 50 μg/ml propidium iodide, and 100 μg/ml RNase A and stained for 30 minutes. After this time the percentage of cells with sub-G_1_ DNA content was determined by flow cytometry.

Microarray analysis. Total mRNA from p65 null and reconstituted MEFs was amplified and labeled with Cyanine 3 (Cy3) or Cyanine 5 (Cy5) dUTP, essentially as described [37] and used for microarray hybridization onto cDNA microarrays. These arrays were manufactured at the NCI Microarray Facility (Frederick, MD) by spotting UniGene mouse cDNA clones (Incyte Genomics) onto glass slides. Data was collected on an Axon scanner where Cy3 and Cy5 fluorescence was measured and compared. Results were expressed as ratio of Cy3 to Cy5 for each experiment. The data shown are averages from 5 independent arrays.

RT-PCR and Northern Blot. Total RNA from p65 null and reconstituted MEFs was obtained using Trizol (Gibco BRL) following the manufacturer's instructions. For RT-PCR 1 μg of RNA was used to generate cDNA using the GeneAmp RNA PCR kit (Perkin Elmer) which was then used to amplify the corresponding genes with specific oligonucleotides. The coding sequence amplified for each gene were; caspase-3, 62-772; caspase-9, 205-1264; GAPDH, 339-865; Bcl-X, 122-487; A1, 133-387, Bcl-w, 65-503, Bok, 50-482; Bak, 31-585, Noxa(CDS), 1-312; Noxa (probe 1), 1-1040; Noxa (probe 2) 1230-1832; Bmf, 81-434; Bad, 109-366; Hrk, 1-242; Bim, 33-306; Bid, 32-292; PUMA, 85-485; and Bik, 120-399.

For Northern blot 10 μg of RNA was loaded per lane onto a 1% agarose-formaldehyde gel. The RNA was transferred to Hybond-N+ membranes (Amersham Pharmacia) and hybridized with [^32^P]-labeled cDNA probes using ExpressHyb Hybridization solution (Clontech) following the manufacturer's instructions. The Noxa probe was generated as a PCR fragment from the mRNA extending from 1 to 1040. PUMA probe was generated by PCR amplification of the 85-485 fragment of mouse mRNA. GAPDH probe was purchased from SeeGene.

Luciferase assays. For reporter assays, Saos-2 p53 Tet-on cells were transfected with 0.5 μg of luciferase reporter and varying amounts of the appropriate expression vector using Lipofectamine Plus (Invitrogen) according to manufacturer's instructions. The Noxa reporter was made placing the -183 to +149 (SacII/SacII) fragment of the murine Noxa promoter [38] in the SmaI site in pGL3-Basic (Promega).  PG13-Luc, containing a generic p53 response element [39] and NF3TK-Luc, containing a trimer of the NF-kB site in the H2-k promoter [40], were also used.  Cells were harvested 48 hr after transfection and luciferase activity was measured in duplicate with the Optocomp II luminometer (MGM Instruments) using 20 μl cell lysate, 100 μl substrate injection and 10 second count time.  Results are expressed as fold induction above control.
